# The molecular basis of lactase persistence: Linking genetics and epigenetics

**DOI:** 10.1111/ahg.12575

**Published:** 2024-08-22

**Authors:** Céleste E. Cohen, Dallas M. Swallow, Catherine Walker

**Affiliations:** ^1^ Department of Genetics, Evolution and Environment University College London Genetics Institute (UGI) London UK; ^2^ Department of Biological Sciences, Graduate School of Science The University of Tokyo Tokyo Japan

**Keywords:** epigenetics, gene expression, lactase persistence, methylation, multiple alleles, transcription factors

## Abstract

Lactase persistence (LP) — the genetic trait that determines the continued expression of the enzyme lactase into adulthood — has undergone recent, rapid positive selection since the advent of animal domestication and dairying in some human populations. While underlying evolutionary explanations have been widely posited and studied, the molecular basis of LP remains less so. This review considers the genetic and epigenetic bases of LP. Multiple single‐nucleotide polymorphisms (SNPs) in an *LCT* enhancer in intron 13 of the neighbouring *MCM6* gene are associated with LP. These SNPs alter binding of transcription factors (TFs) and likely prevent age‐related increases in methylation in the enhancer, maintaining *LCT* expression into adulthood to cause LP. However, the complex relationship between the genetics and epigenetics of LP is not fully characterised, and the mode of action of methylation quantitative trait loci (meQTLs) (SNPs affecting methylation) generally remains poorly understood. Here, we examine published LP data to propose a model describing how methylation in the *LCT* enhancer is prevented in LP adults. We argue that this occurs through altered binding of the TF Oct‐1 (encoded by the gene *POU2F1*) and neighbouring TFs GATA‐6 (*GATA6*), HNF‐3A (*FOXA1*) and c‐Ets1 (*ETS1*) acting in concert. We therefore suggest a plausible new model for *LCT* downregulation in the context of LP, with wider relevance for future work on the mechanisms of other meQTLs.

## 1 INTRODUCTION

Lactose is a carbohydrate present in most mammalian milks and is a primary energy source for neonates before weaning. It is a disaccharide composed of two sugar molecules, glucose and galactose, linked by a β‐1,4 glycosidic bond. Young mammals express the β‐galactosidase enzyme lactase‐phlorizin hydrolase (LPH) which hydrolyses the glycosidic bond, allowing glucose and galactose to be absorbed into the small intestinal enterocytes — absorptive epithelial cells lining the intestinal wall — and into the bloodstream. LPH is expressed at the brush border membrane of the enterocytes (Naim et al., 1987; Smith et al., 1985). While LPH activity is high in neonates, it rapidly declines in most mammals during or after weaning. In humans, this decrease is called lactase non‐persistence (LNP). The progressive decline of LPH in LNP individuals can be first detected from approximately 2 years of age, often causing symptoms of lactose intolerance after milk consumption. This decline has been attributed to downregulation of the *LCT* gene, which encodes LPH (Wang et al., 1998). However, in some 35% of humans worldwide, lactase expression persists into adulthood (Itan et al., 2009).

With earliest domestications of sheep, cows and goats by humans ∼10,000 years ago (Zeder, 2008), some human populations began incorporating animal milk into their diets. This shift in diet is associated with the spread of lactase persistence (LP) (Ségurel & Bon, 2017). LP has been linked to genetic variants near *LCT*, which are associated with strong signatures of recent positive selection (Bersaglieri et al., 2004; Coelho et al., 2005; Jones et al., 2013; Sabeti et al., 2007; Tishkoff et al., 2007). Based on high levels of linkage disequilibrium around *LCT* and allele frequency data, selection coefficients for LP have been estimated at ∼3%–19% in some Northern European populations (Bersaglieri et al., 2004; Coelho et al., 2005; Tishkoff et al., 2007), representing some of the strongest signals of recent positive selection documented in humans, and resulting in its worldwide distribution seen today (Itan et al., 2009; Liebert et al., 2017). Additionally, LP has been associated with obesity and body mass index (BMI) (Albuquerque et al., 2013; Almon et al., 2012; de Luis et al., 2021; Hartwig et al., 2016; Lamri et al., 2013), although covariates such as lifestyle, nutrition or microbiome composition and population stratification may confound such associations. While the study of LP has relevance beyond evolutionary biology to health and gastroenterological conditions, its molecular mechanisms are poorly understood.

The genetic basis of LP has been relatively well studied across different populations, and recent research has uncovered epigenetic variation characteristic of LP. Specifically, the most studied ‘European’ LP‐associated single‐nucleotide polymorphism (SNP) is believed to impact DNA methylation (see Box 1) near *LCT* and cause LP (Labrie et al., 2016). This review examines published data on lactase regulation together with LP genetics and epigenetics. We propose an explanation involving transcription factors (TFs) to explain the molecular mechanisms linking LP genetics and epigenetics. This model may have wider relevance in understanding temporal epigenetic dynamics and epigenetic heritability underlying other human phenotypes and diseases.

**DNA methylation and meQTLs**

DNA methylation involves DNA methyltransferase (DNMT) adding methyl groups to cytosine 5′‐carbons at CpG dinucleotides (Miller & Grant, 2013), which tend to cluster in promoters, enhancers and silencers. Their methylation can influence gene regulation by affecting accessibility of DNA to transcription machinery and other regulatory proteins. Hypermethylation (increased methylation) tends to impair transcription factor (TF) binding to regulatory regions and downregulate genes, as opposed to hypomethylation (decreased methylation) which is associated with gene activation (Miller & Grant, 2013). SNPs that are associated with changes in methylation, as found in LP, have been termed methylation quantitative trait loci (meQTLs) (Villicaña & Bell, 2021). meQTLs have been identified across a number of diseases including type 2 diabetes (Xue et al., 2018) and Parkinson's disease (Pihlstrøm et al., 2015). However, meQTLs and the mechanisms by which they directly or indirectly affect methylation remain understudied. These mechanisms can vary across diseases and genetic regions, impeding a consensus understanding, but the current leading hypothesis is that altered binding of TFs affects methylation and demethylation dynamics (Villicaña & Bell, 2021).

## 2 GENETICS OF LP

### 2.1 An enhancer 14‐kb upstream of *LCT*

**FIGURE 1:**
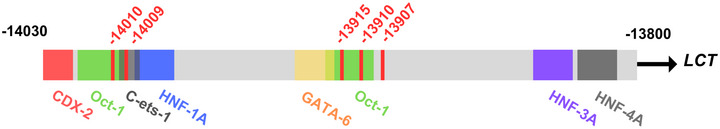
Schematic *MCM6* intron 13 lactase persistence (LP) enhancer region as identified by Troelsen et al. (2003). The light grey box with coloured segments represents the sequence from −13,800 bp (GRCh38 chr2:135,850,966) to −14,030 bp (GRCh38 chr2:135,851,196) of *LCT*. The coloured boxes and corresponding annotations are transcription factor binding sites, and the red lines and annotations are LP‐associated SNPs with their positions (not to scale) (Jensen et al., 2011; Lewinsky et al., 2005; Liebert et al., 2016).

The *LCT* gene spans 49,336 nucleotides on the reverse strand of human chromosome 2q21 and is flanked by the genes *UBX4* and *MCM6*. By studying expression patterns of polymorphic *LCT* transcripts in European individuals, LP‐associated LPH expression was determined as mediated by cis‐acting variation and characterised by an autosomal codominant pattern of inheritance, with sucrase‐normalised LPH activity showing a trimodal distribution across individuals (Box 2) (Ho et al., 1982; Wang et al., 1998). Variant alleles in an enhancer in intron 13 of *MCM6* are responsible for preventing or bypassing *LCT* downregulation in adulthood, causing LP. Five SNPs have been identified as significantly associated with LP and functionally shown to affect *LCT* regulation: *−13910C>T* (rs4988235), *−13907C>G* (rs41525747), *−13915T>G* (rs41380347), *−14010G>C* (rs145946881) and *−14009T>G* (rs869051967) (Figure 1) (Enattah et al., 2002; Ingram et al., 2007, 2009; Jones et al., 2013; Poulter et al., 2003; Tishkoff et al., 2007). These have been identified separately in different populations worldwide and on different genetic backgrounds. Studying their effects on enhancer activity and identifying putative binding TFs have provided insight into possible genetic mechanisms of LP.

**The genetics of lactase persistence (LP)**

LP is caused by SNPs in intron 13 of the adjacent upstream gene *MCM6*: LP‐associated SNPs act co‐dominantly on lactase mRNA and enzyme levels, giving intermediate levels in heterozygous adults. However, individuals who carry only one LP‐associated allele express sufficient levels of lactase to digest the lactose load in a lactose‐tolerance test, usually without gastrointestinal symptoms. LP is thus often considered a dominant phenotype.

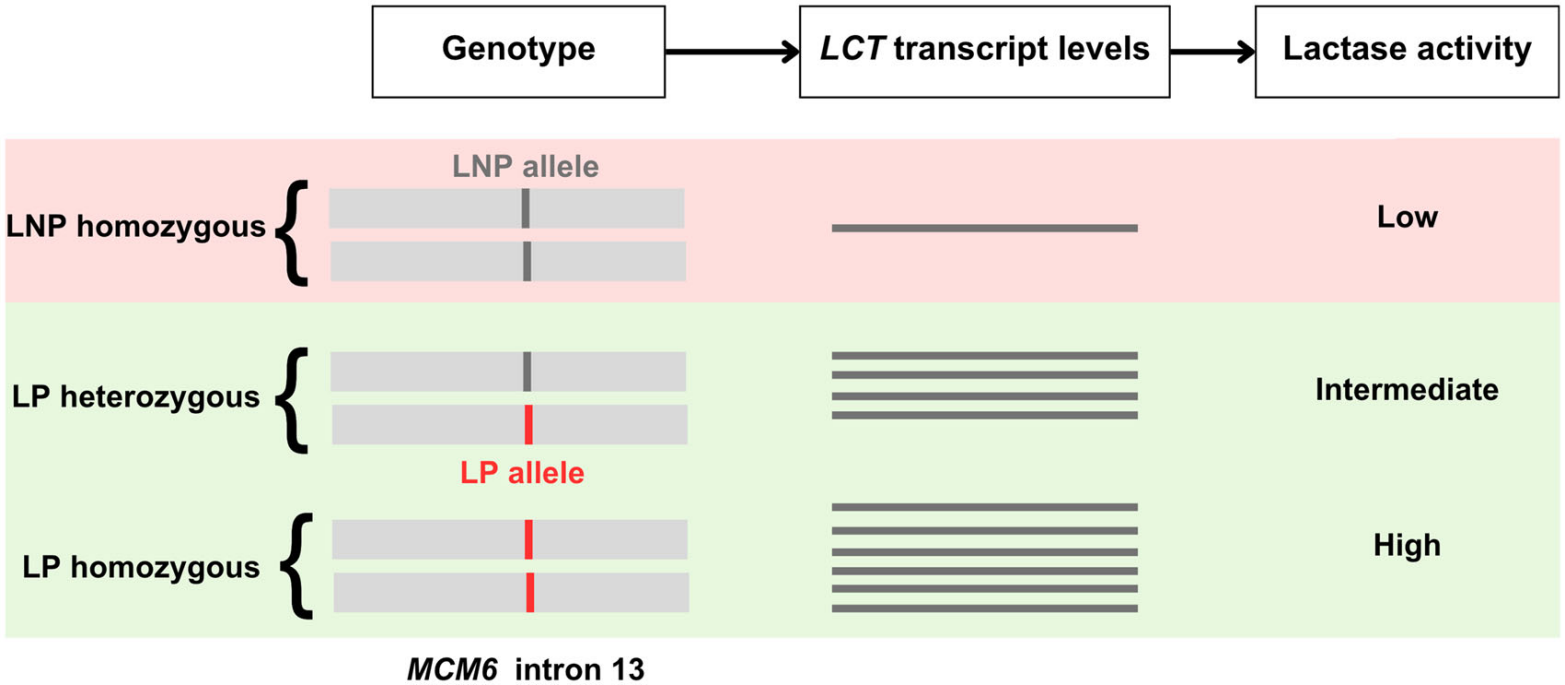

### 2.2 A common European SNP: −13910C>T

The first and most widely studied LP‐associated SNP was *−13910C>T*, a C>T variant 13,910 bases upstream of the *LCT* transcription start site in a putative enhancer in intron 13 of *MCM6*. It was first identified in Finnish individuals (Enattah et al., 2002) and later in other Northern European populations (Poulter et al., 2003). Further studies have explored the effects of the SNP on *LCT* expression. The T variant increases *LCT* promoter activity in promoter–enhancer luciferase expression constructs in vitro (Lewinsky et al., 2005) in the colon carcinoma cell line Caco‐2. While these short‐term in vitro experiments may not wholly reflect the same cellular environment as the small intestine, a similar effect has also been seen in a mouse model in vivo (Fang et al., 2012), providing evidence of long‐term effects of the SNP on *LCT* expression.

To understand how the enhancer might affect *LCT* expression, further experiments investigating TF binding in the region identified Oct‐1 as the main TF binding at the *−13910C>T* locus (Lewinsky et al., 2005) (Figure 1). Oct‐1 shows greater binding to the T allele compared with the ancestral C allele (Lewinsky et al., 2005) (Figure 1), which can be competed out by both the classical and non‐classical binding motifs. Oct‐1 is known to affect gene regulation in epithelial cells, notably in the intestinal epithelium (Vázquez‐Arreguín & Tantin, 2016).

Other intestinally expressed TFs that also bound nearby sites included GATA‐6, CDX‐2, HNF‐3A and HNF‐4A (Figure 1), and disruptive mutations in all but the CDX‐2 binding site abolished enhancer activity. Overexpression of GATA‐6, HNF‐4A and HNF‐3A in Caco‐2 cells increased enhancer activity but decreased differences in enhancer activity between the −13910*C and *T* alleles (Lewinsky et al., 2005). Amongst these studied TFs, Oct‐1 overexpression had the greatest effect on enhancer activity and did not reduce the difference between the alleles. However, this effect only occurred when co‐transfected with HNF‐1A, a TF known to interact with Oct‐1 (Ishii et al., 2000) and to bind the *LCT* promoter and enhancer (Jensen et al., 2011). Together with observations that sequences upstream of *−13910C>T* are essential to enhance promoter activity (Jensen et al., 2011), these results point towards the involvement of Oct‐1 in mediating *LCT* enhancer activity via interactions with surrounding TFs or TF‐binding sites. Additionally, the link between LP‐associated *−13910C>T* allelic variation, Oct‐1 binding and enhancer activity suggests the involvement of Oct‐1 in LP.

### 2.3 SNPs frequent in Africa and the Middle East

**FIGURE 2:**
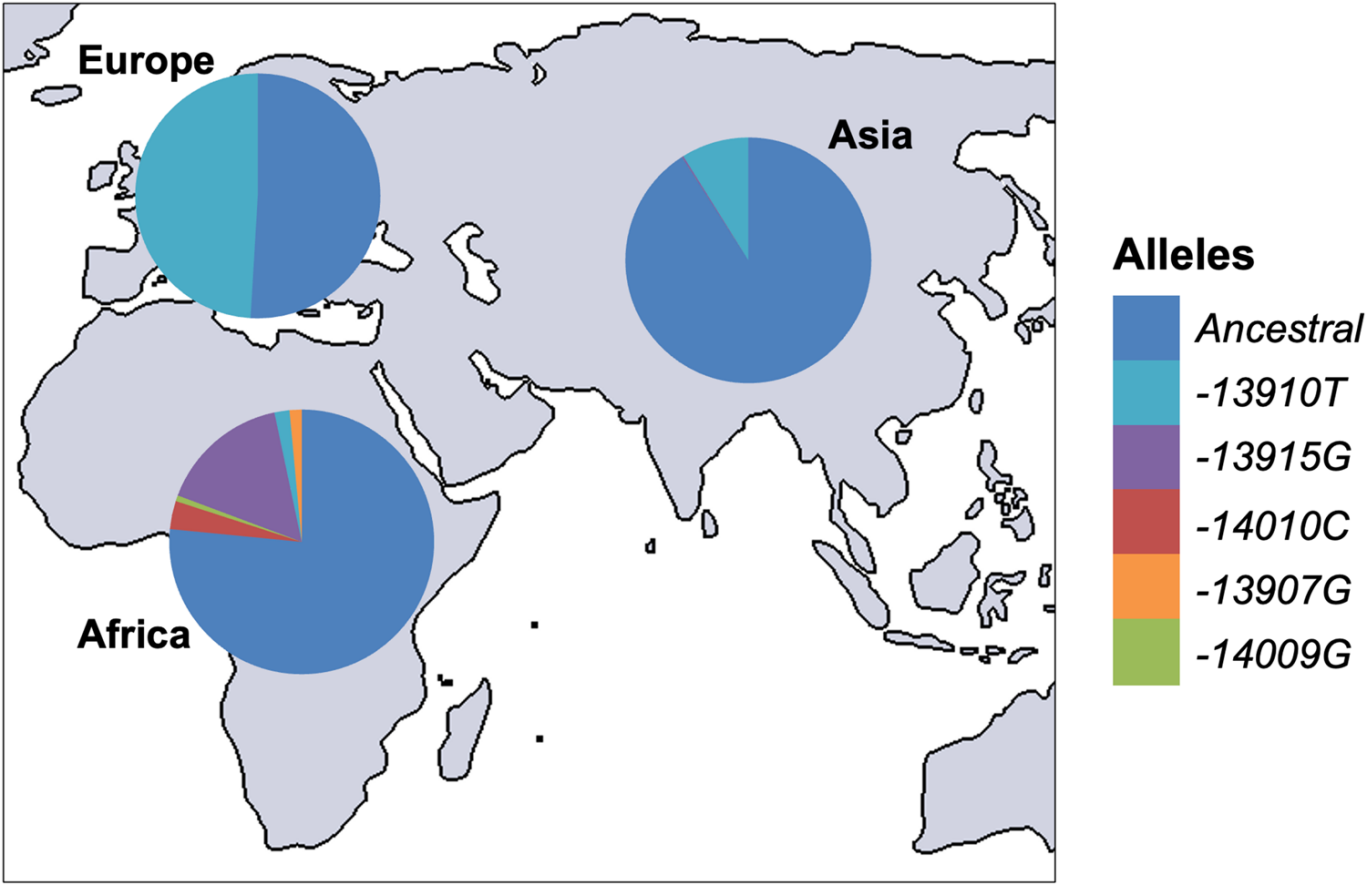
Known lactase persistence (LP) alleles and frequencies in Europe, Asia and Africa. These represent an estimation of allele frequencies in each continent based on samples from different countries. The African pie chart also includes populations from the Arabian Peninsula. Adapted from Swallow (2015).

The −13910*T variant is absent in many lactase‐persistent individuals worldwide, particularly in Africa (Liebert et al., 2017; Mulcare et al., 2004). Four other SNPs are associated with LP and experimentally linked to *LCT* regulation. These have been identified in various populations and on separate haplotypes (Ingram et al., 2022; Liebert et al., 2017; Tishkoff et al., 2007) (Figure 2) and provide support for the involvement of Oct‐1 in mediating LP as well as insights into possible alternative LP mediators.

Two LP‐associated variants −*13907C>G* and *−13915T>G* (Ingram et al., 2007, 2009; Jones et al., 2013; Tishkoff et al., 2007) are adjacent to (*−13907C>G*) or within (*−13915T>G*) the known Oct‐1 binding site (Figure 1). Like *−13910C>T*, they seem to increase Oct‐1 binding, although somewhat variably across studies (Enattah et al., 2008; Ingram et al., 2007; Olds et al., 2011), and both increase enhancer activity in vitro (Tishkoff et al., 2007). Upstream of *−13910C>T*, *−14010G>C* is located in a second Oct‐1 binding site and near an HNF‐1A binding site (Figure 1). Here, the C allele increases binding of Oct‐1 only in the presence of the adjacent HNF‐1A motif (Jensen et al., 2011; Tishkoff et al., 2007). −14010*C also increases enhancer activity to a similar extent to −13910*T. Finally, a −14009*G variant increases *MCM6* intron 13 enhancer activity compared with its ancestral T variant (Ingram et al., 2009; Jones et al., 2013), showing similar enhancing activity to −13910*T (Liebert et al., 2016). However, instead of being bound by Oct‐1, −14009*G is bound by C‐ets‐1 (Liebert et al., 2016) (Figure 1). Ets (E26 transformation‐specific) TFs are known to play a role in DNA regulation by interacting with other proteins (Findlay et al., 2013) and are notably shown to commonly colocalise with Oct‐1 (Song et al., 2021). It is possible that C‐ets‐1 binding affects Oct‐1‐mediated enhancer activity through colocalisation or may alter regulation independently.

While the commonly studied −13910*T variant has been shown to maintain long‐term *LCT* promoter activity in vivo, in transgenic mice, the effect of the four other LP‐associated SNPs has yet to be shown this way. These four LP‐associated SNPs show the same characteristics as *−13910C>T*: they are all associated with LP, affect *LCT* enhancer activity, are in the immediate vicinity of an Oct‐1 binding site and alter the binding of Oct‐1 or a possibly associated TF C‐ets‐1. This indicates convergent evolution, where variants affecting binding of Oct‐1 at different loci, or the associated TF C‐ets‐1, arose multiple times in different haplotypes, suggesting an instrumental role of Oct‐1 binding in LP.

### 2.4 Oct‐1—a central mechanism of LP?

Oct‐1 binding has thus been identified as a likely mediator of LP. Its putative interactions with HNF‐1A and predicted binding sites of GATA‐6, CDX‐2, HNF‐3A and HNF‐4A (Figure 1) (Lewinsky et al., 2005), as well as the importance of regions throughout the enhancer in modulating its activity (Jensen et al., 2011), suggest that its functional *LCT*‐regulatory role depends on multiple protein–protein and protein–DNA interactions. However, how Oct‐1‐mediated *LCT* upregulation relates to LP requires further investigation. Regulatory assays do not explain the temporal effects of LP‐associated SNPs and Oct‐1 binding.

## 3 EPIGENETICS OF LP

While genomes remain stable and relatively unchanged throughout a lifespan, epigenetic modifications change across tissues and through time and are increasingly recognised as playing a pivotal role in gene regulation (Miller & Grant, 2013; Wang et al., 2022). Recent research has provided evidence for an epigenetic basis of the temporal nature of LP, with a focus on effects of the −13910*T LP‐associated allele on CpG methylation (Labrie et al., 2016; Leseva et al., 2018; Oh et al., 2017).

Labrie and colleagues (2016) initially assessed the relationship between epigenetic DNA modifications and *LCT* transcript levels in jejunal samples from a cohort of people from Lithuania. CpG methylation levels were fine‐mapped throughout the *LCT–MCM6* genetic region and compared with steady‐state *LCT* mRNA levels (Labrie et al., 2016). This revealed a significant inverse correlation between *LCT* expression and modified CpG sites in the *MCM6* intron 13 enhancer and exon 16 in enterocytes, suggesting their importance in epigenetically modulating LP. Comparison of CpG methylation across *LCT–MCM6* between groups of individuals with different *−13910C>T* genotypes also revealed lower methylation levels in T allele‐carrying individuals at sites in *MCM6* intron 13 and exon 16, with the most marked differences for intron 13 (Labrie et al., 2016). The detection of intron 13 supports its involvement in LP and the role of *−13910C>T*. It is interesting that *MCM6* exon 16 methylation also differs between *−13910C>T* genotypes. Based on ENCODE predictions (from data generated by the ENCODE Data Analysis Center) (ENCODE Project Consortium et al., 2020), the region also overlaps with a putative enhancer. Further research is needed to determine the importance of exon 16 in LP and its impact on *LCT* expression and whether the observed −13910*T dosage‐related differential methylation is incidental or functional. It is striking here that this SNP (a methylation quantitative trait locus [meQTL]) is affecting methylation locally in intron 13 and also over 5‐kb away in exon 16.

Hypomethylation of the intron 13 enhancer in adult TT homozygotes (and intermediate levels in CT heterozygotes) is consistent with its suggested role in maintaining *LCT* expression in the jejunum of LP individuals. This hypomethylation is likely causal of the genetically determined differences in adult lactase expression. In non‐adult hospital patients of European ancestry, better correlation is seen between the level of methylation and lactase activity than with genotype (Leseva et al., 2018).

These findings overall suggest that the −13910*T allele in *MCM6* intron 13 enhancer causes genetically determined LP by modulating enhancer methylation. Further in vitro studies in Caco‐2 cells or intestinal organoids via epigenetic editing techniques such as a modified CRISPR‐Cas9 system (Kang et al., 2019) would clarify how *−13910C>T* acts as an meQTL for LP.

## 4 EVALUATING LP GENETIC AND EPIGENETIC INTERACTIONS IN RELATION TO LP

There is currently a lack of focused research on how genetic variation can affect DNA methylation profiles, with some exceptions (Villicaña & Bell, 2021). Existing models may help understand the mechanisms of meQTLs associated with LP which, in turn, could serve as a model to study these molecular dynamics.

Both in mice (Maegawa et al., 2010) and humans (Kane & Sinclair, 2019) alike, DNA methylation patterns shift significantly as organisms develop and age, tending towards hypermethylation. In the context of *LCT*, the differences in methylation appear to be tightly regulated in a tissue‐ and genomic‐region‐specific manner, unlike the natural hypermethylation of DNA with age. The question of how meQTLs such as LP‐associated SNPs affect age‐related methylation profiles in this tissue‐ and region‐specific manner remains. A leading hypothesis is that TF binding can affect methylation of nearby CpG islands by influencing methylating/demethylating enzymatic activity (Banovich et al., 2014; Villicaña & Bell, 2021). Altered binding caused by different alleles may modify this process, in turn impacting methylation. Two groups of enzymes regulate methylation: DNA methyltransferases (DNMTs), which cause methylation by adding methyl groups onto cytosine 5′‐carbons, and TET enzymes, which reverse methylation by oxidising 5‐methylcytosine (Wu & Zhang, 2017). Although research on TF effects on methylation remains sparse, it is known that some TFs affect methylation by occupying genomic sites in a way that passively prevents their methylation or demethylation, as observed by the SOX2 TF inhibiting DNA methylation maintenance by the DNMT1 enzyme during replication (Vanzan et al., 2021). Alternatively, some TFs recruit enzymes that actively alter methylation, as seen in the regulation of genes involved in the DNA damage response, bound by the c‐Myc TF which recruits the TET2 enzyme to demethylate and upregulate target genes (Chen et al., 2018).

In the context of LP, it is likely that the −13910*T allele, and possibly other LP‐associated variants, reverses or prevents age‐related methylation in *MCM6* intron 13, which causes LNP via the action of bound TFs. As previously discussed (see Section 2), published data suggest that Oct‐1 may play a pivotal role in the process: it is the main TF identified as binding to the *−13910C>T* locus, and its binding is the main parameter that has been found to differ by genotype (Lewinsky et al., 2005). Additionally, altered Oct‐1 binding seems to be common to most LP‐associated SNPs. While Oct‐1 binding alone has not been shown to alter methylation, it could affect neighbouring TFs which have been shown to do so. A study that bioinformatically predicted and experimentally validated demethylation‐associated TFs identified only 28 of 383 TFs (∼7%) as promoting demethylation around their binding sites (Miyajima et al., 2022). Interestingly, 3 of the 7 TFs (∼43%) shown to bind to the *MCM6* intron 13 enhancer in LP‐related studies (Figure 2) featured among this list of 28 ‘demethylating’ TFs: HNF‐3A, GATA‐6 and C‐ets‐1. In multiple studies (Lemma et al., 2022; Suzuki et al., 2022), HNF‐3A and GATA‐6 have been shown to interact with demethylating TET enzymes, hence causing active demethylation. In the context of the intron 13 enhancer, they likely also play a role in Oct‐1‐mediated enhancer activity, suggesting a level of collaboration with Oct‐1 (Lewinsky et al., 2005). Similarly, it is known that C‐ets‐1 commonly colocalises, and possibly interacts, with Oct‐1 (Song et al., 2021).

**FIGURE 3:**
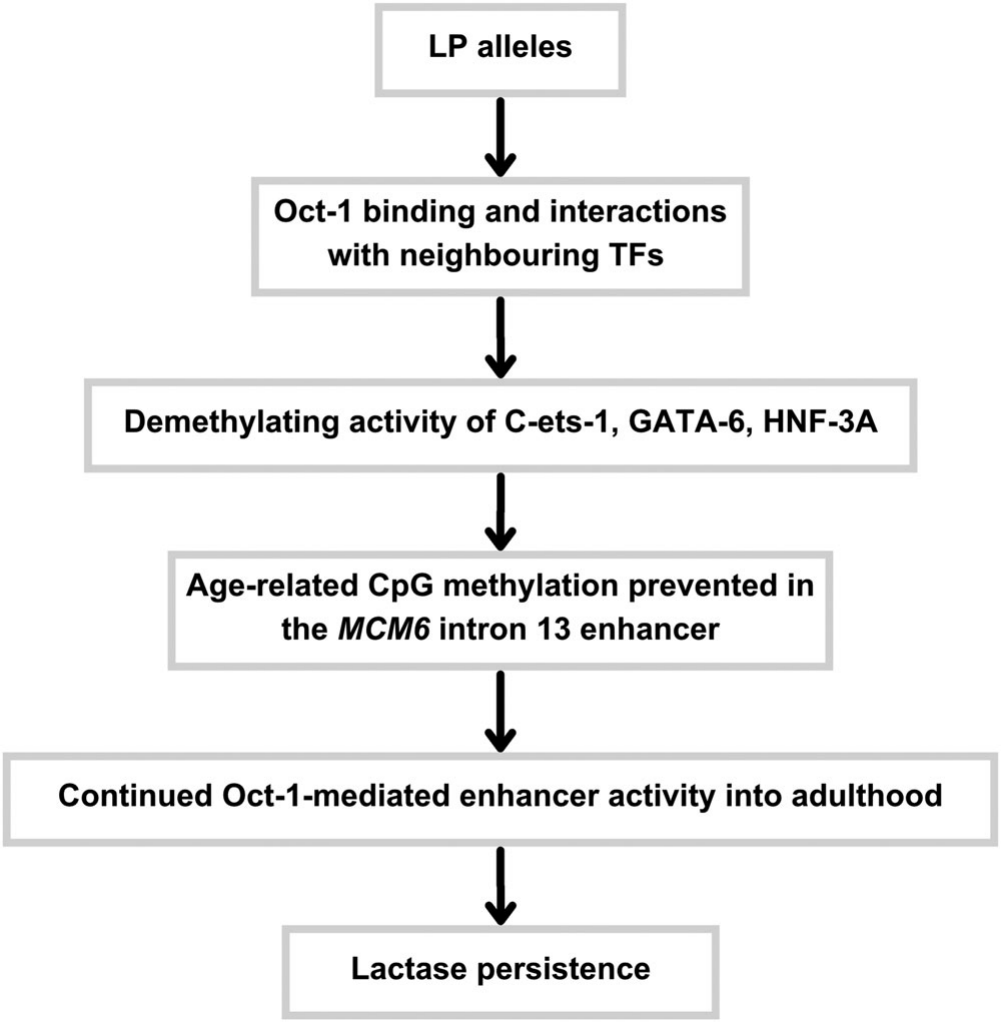
Flowchart of proposed genetic and epigenetic mechanisms causing lactase persistence (LP). LP‐associated alleles enhance Oct‐1 binding and possible interactions with demethylating transcription factors (TFs) such as C‐ets‐1, GATA‐6 and HNF‐3A. These likely act against age‐related methylation in the *LCT* enhancer, allowing for continued Oct‐1‐mediated enhancer activity and LP.

It is thus plausible that the demethylating activity of GATA‐6, C‐ets‐1 and HNF‐3A reverses or prevents LP enhancer methylation seen in LNP adults, causing LP (Figure 3). As they were not identified as binding to the *−13910C>T* SNP like Oct‐1 (Lewinsky et al., 2005), the latter could promote the demethylating activity of its neighbouring TFs. This type of effect has previously been observed between other TFs. For instance, SOX‐2‐mediated demethylation increases in vitro when interacting with non‐demethylating Oct‐4/POU5F1 (Vanzan et al., 2021).

It is important to note that Oct‐1 binding can also be inhibited by CpG methylation (Murayama et al., 2006). Assuming that the enhancer shows low methylation in neonates, Oct‐1 can still bind. In LP individuals, high Oct‐1/C‐ets‐1 binding to the unmethylated enhancer in infants could prevent its methylation after weaning via adjacent demethylating TFs. This would allow continued Oct‐1 binding and consequent enhancer activity into adulthood (Figure 3). In LNP individuals, methylation would increase, eventually inhibiting Oct‐1 binding. Further research is needed to validate the proposed model and better understand the complex mechanisms that are causative of LP.

### 4.1 Chromatin looping and demethylation

In their adult‐only study, Labrie and colleagues (2016) noted that methylation changed over time across multiple regions beyond the LP enhancer (*MCM6* intron 13), including the *LCT* promoter, *LCT* intron 2 and *MCM6* exon 16. These age‐related changes differed across *−13910C>T* genotype, despite being observed in patients over 21 years old (by which age, LNP adults only express low amounts of lactase). −13910*TT (LP) homozygous adults showed generally decreasing methylation with age across the *LCT–MCM6* region, whereas the opposite was seen in −13910*CC (LNP) individuals. While these differences were only slight for the promoter and intron 2—two cell‐specific hypomethylated regions whose methylation did not differ significantly by genotype, unlike *MCM6* exon 16—they suggest that differential methylation likely caused by demethylating TFs could extend beyond the *MCM6* intron 13 enhancer region, marginally affecting other *LCT*‐regulatory sites over time.

**FIGURE 4:**
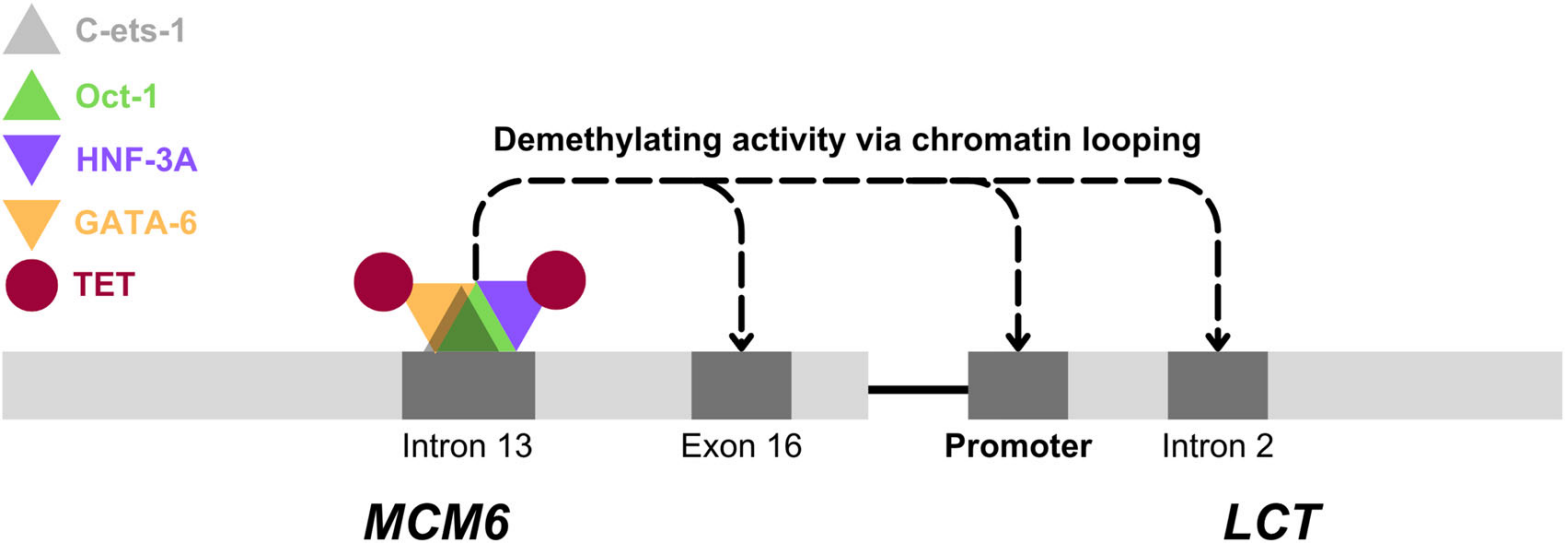
Schematic of putative *LCT–MCM6* methylation‐altering activity of transcription factors (TFs) bound to *MCM6* intron 13 via chromatin looping in lactase persistence (LP) individuals. Light and dark grey boxes represent the *MCM6* and *LCT* genes; dark grey regions represent regions differentially methylated with age between LP and lactase non‐persistence (LNP) individuals (specifically −13910*TT and *CC* homozygotes). The black connecting line is the intergenic region. Coloured triangles are TFs, which all bind at *MCM6* intron 13 in LP individuals and carry out demethylating activity partly via GATA‐6 and HNF‐3A recruitment of TET demethylating enzymes shown as red circles and slight demethylating activity shown in black dashed arrows.

The overall decrease in methylation of the promoter, intron 2 and *MCM6* exon 16 in LP individuals with age might occur through interactions between LP enhancer‐binding demethylating TFs with distal genetic regions through chromatin looping (Figure 4) (Villicaña & Bell, 2021). *MCM6* exon 16 and *LCT* intron 2 are likely *LCT* enhancers. In fact, CRISPR‐Cas9 deletion of part of intron 2 caused significantly lower lactase mRNA levels in mice (Labrie et al., 2016), and ENCODE predictions identify a putative enhancer in exon 16 (ENCODE Project Consortium et al., 2020). Additionally, these regions are hypomethylated in enterocytes, supporting their role in lactase regulation (Labrie et al., 2016). Therefore, these regions likely come into contact with the *LCT* promoter via chromatin looping to exert enhancer activity, alongside the LP enhancer in *MCM6* intron 13. This could cause the promoter, *MCM6* exon 16 and *LCT* intron 2 to lose methylation through contact with the *MCM6* LP enhancer‐bound demethylating TFs in LP adults. While this region‐wide demethylation is not primarily causative of LP, the distal effects of demethylating TFs via chromatin looping are under‐researched and worth investigating. Here, LP is an apposite example of both local and distant effects of a single meQTL in a sequence‐based characterisation of methylation profiles within a large genetic region (Labrie et al., 2016). Visualising chromatin loops in the region via methods such as Hi‐C (Ron et al., 2017) could help investigate these hypotheses.

## 5 FURTHER CONSIDERATIONS

In LP individuals of 13910*TT genotype, an *LCT* antisense non‐coding RNA (asRNA) gene shows increased DNA methylation over time, unlike the rest of the *LCT–MCM6* region. Studying trends in methylation of the asRNA promoter and *LCT* mRNA levels has shown that both increased methylation and expression of the asRNA were associated with increased *LCT* mRNA levels (Labrie et al., 2016). An insulator CTCF‐binding motif is predicted by ENCODE to overlap the asRNA promoter (ENCODE Project Consortium et al., 2020), where methylation may inhibit CTCF binding (Kornblihtt, 2012). This could allow for stronger interactions between the asRNA promoter and nearby enhancers, explaining higher asRNA expression levels given increased methylation. The asRNA could then act to increase *LCT* transcription or interact with *LCT* mRNA and support mRNA stability or processing (Villegas & Zaphiropoulos, 2015).

Given that increased methylation of the asRNA promoter is positively correlated with increased *LCT* expression, it logically follows that LP individuals would have higher levels of asRNA methylation. It is also consistent with our model that asRNA methylation does not decrease over time in LP individuals like *LCT* enhancers, because it is unlikely to bind to the *LCT* promoter, thus avoiding the LP enhancer demethylating TFs. However, it would be interesting to investigate how *TT* genotype, which seems to promote demethylation across the rest of the *LCT–MCM6* region, is associated with increased methylation around the asRNA.

Additionally, mosaicism of lactase expression in enterocytes has been observed in some LNP individuals, with some cells expressing lactase and some not (Maiuri et al., 1991). This could be due to stochasticity in the molecular mechanisms causing epigenetic changes in the *LCT–MCM6* region or even in Oct‐1 expression levels. Simulations have shown that collaborative binding of different TFs and overlap of binding sequences can introduce noise in transcription (Parab et al., 2022). The partial overlap of multiple binding sites in the *MCM6* intron 13 enhancer (Figure 1) could lead to stochasticity in binding and subsequent demethylating activity. Oct‐1 binding could simply increase the likelihood that the *MCM6* intron 13 enhancer remains unmethylated, rather than consistently promoting demethylation, resulting in some variation between cells despite overall discrete LP/LNP phenotypes. Other parts of the proposed processes underlying *LCT–MCM6* methylation changes, such as TET recruitment by demethylating TFs, could also introduce variation resulting in mosaicism, but overall, the source of mosaicism requires further investigation.

In the longer term, it will be interesting to understand both how and why lactase expression is normally so tightly developmentally regulated in all mammals. While CpG island hypermethylation occurs genome‐wide with age (Zampieri et al., 2015), enterocyte hypermethylation causing LNP apparently occurs during childhood and in a sequence‐specific manner. Sequence‐specific de novo methylation, as seen in LNP, is typically thought to stem from DNMT recruitment by DNA‐binding proteins such as TFs or polycomb proteins (Hervouet et al., 2018), but these remain to be identified for LNP. Finally, the fundamental question of why lactase is normally downregulated in mammals remains. There have been suggestions including maternal health, birth spacing and the role of lactase in hydrolysing other potentially toxic glycosides such as phlorizin. In addition to researching the environmental and cultural drivers of this phenomenon, further work is merited to explore the molecular mechanisms of lactase downregulation across species, for which animal models may be suitable.

## 6 CONCLUSIONS AND FUTURE DIRECTIONS

This review has provided evidence that:

1. Oct‐1 binding to the *MCM6* intron 13 enhancer is central to enhancer activity and likely an important mechanism by which LP‐associated variants prevent lactase downregulation.
2. Low methylation in the enhancer, due to LP‐associated variants, allows for continued expression of *LCT* into adulthood.
3. Increased Oct‐1 binding at the enhancer due to LP‐associated variants could prevent local methylation via interactions with neighbouring TFs, working against LNP age‐associated methylation to maintain *LCT* expression over time.
4. Methylation in adults beyond the LP enhancer appears to slightly change with age by genotype, possibly due to demethylating effects spreading via chromatin looping. We highlight how a single SNP can affect methylation across multiple genetic regions.

This review has also synthesised recent studies to posit that the surrounding demethylating TFs GATA‐6, HNF‐3A and C‐ets‐1 are likely key mediators of the epigenetic effect of *−13910C>T*, here classifiable as an meQTL. We propose a possible new model for *LCT* regulation in the context of LP, which has wider relevance for future work on the mechanisms of other meQTLs. Experimental validation, possibly by identifying how and when methylation regulators (DNMT and TET enzymes) act on the enhancer, and whether they form complexes with these TFs, would be necessary to confirm this model. Additionally, studying putative interactions between Oct‐1 and surrounding TFs or their binding sequences and visualising small‐scale interactions between different parts of the enhancer through high‐resolution imaging techniques (reviewed in Price et al., 2021) may help test these hypotheses.

Some specific questions surrounding LP still require further research. Most of our knowledge comes from the *−13910C>T* variant, reflecting a Eurocentric research bias. Investigating other LP‐associated variants in more detail is warranted and may yield further insights. Additionally, the study of small intestinal methylation for the *LCT–MCM6* region in foetuses, infants and adolescents would help confirm the role of methylation changes in LP, as it has been assumed but not verified that the LP enhancer is hypomethylated in all healthy children in whom lactase expression is high. Lastly, some of the perceived phenotypic variation in lactose tolerance across individuals is likely due to differences in gut microbiomes, as milk‐consuming LNP individuals (homozygous for the C allele) show increases in the lactose digester *Bifidobacterium* (Bonder et al., 2016; Kurilshikov et al., 2021), and is also related to differences in food transit times which positively correlate with lactose digestion (Labayen et al., 2001). As the gut microbiome has been shown to affect intestinal stem cell gene methylation patterns in a complex, directed way during development (Yu et al., 2015), it may even play a role in epigenetic regulation itself.

## AUTHOR CONTRIBUTIONS

Céleste Cohen researched and wrote the manuscript and produced the figures. Catherine Walker provided supervision and manuscript development, editing and preparation for publication. Dallas Swallow contributed to conceptualisation and editing of this work. All authors approved the submitted version.

## CONFLICT OF INTEREST STATEMENT

The authors declare no conflicts of interest.

## Data Availability

Data sharing not applicable to this article as no datasets were generated or analysed during the current study.
